# Liver CT for vascular mapping during radioembolisation workup: comparison of an early and late arterial phase protocol

**DOI:** 10.1007/s00330-016-4343-1

**Published:** 2016-04-23

**Authors:** Andor F. van den Hoven, Manon N. G. J. A. Braat, Jip F. Prince, Pieter J. van Doormaal, Maarten S. van Leeuwen, Marnix G. E. H. Lam, Maurice A. A. J. van den Bosch

**Affiliations:** Department of Radiology and Nuclear Medicine, University Medical Center Utrecht, Heidelberglaan 100, 3584 CX Utrecht, The Netherlands

**Keywords:** Radioembolisation, SIRT, Liver CT, Acquisition protocol, Arterial phase

## Abstract

**Objectives:**

To compare right gastric (RGA) and segment 4 artery (A4) origin detection rates during radioembolisation workup between early and late arterial phase liver CT protocols.

**Methods:**

100 consecutive patients who underwent liver CT between May 2012–January 2015 with early or late arterial phase protocol (n = 50 each, 10- vs. 20-s post-threshold delay) were included. RGA/A4 origin detection rates, assessed by two raters, and contrast-to-noise ratio (CNR) of the hepatic artery relative to the portal vein were compared between the protocols.

**Results:**

The first–second rater scored the RGA origin as visible in 58–65 % (specific proportion of agreement 82 %, κ = 0.62); A4 origin in 96–89 % (94 %, κ = 0.54). Thirty-six percent of RGA origins not detectable by DSA were identified on CT. Origin detection rates were not significantly different for early/late arterial phases. Mean CNR was higher in the early arterial phase protocol (1.7 vs. 1.2, *p* < 0.001).

**Conclusion:**

A 10-s delay arterial phase CT protocol does not significantly improve detection of small intra- and extrahepatic branches. RGA origin detection requires further optimization, whereas A4/MHA origin detection is adequate, with good inter-rater reproducibility. CT remains important for preprocedural planning, because it may reveal arterial anatomy not discernible on DSA.

***Key Points*:**

• *An early arterial phase does not significantly improve RGA and A4/MHA origin detection.*

• *RGA origin detection (58*–*65 %) on CT is still suboptimal.*

• *36 % of RGA origins undetectable on DSA can be identified on CT.*

• *A4/MHA origin detection (89*–*96 %) on CT is excellent.*

• *Inter-rater reproducibility is good for RGA and A4/MHA origin detection on CT.*

## Introduction

Radioembolisation has evolved as a safe and effective treatment option in patients with liver tumours that are not resectable and are refractory to standard systemic therapy [1]. Since this treatment encompasses the injection of radioactive microspheres via a microcatheter positioned in the hepatic arteries, it is essential to perform a thorough assessment of the hepatic arterial anatomy before treatment [2].

Intra-procedural imaging with digital subtraction angiography (DSA) has long been considered the gold standard for vascular evaluation [3]. However, since modern multidetector computed tomography (CT) scanners enable high-resolution, multiphasic, multiplanar imaging of the liver, the arterial vasculature can already be evaluated on CT before the pretreatment angiography. This has two distinctive advantages. First, in contrast to DSA, CT images can depict the spatial relation between arterial branches and liver parenchyma or gastrointestinal organs in three dimensions, at high resolution, with a wide field of view. Most importantly, though, timely assessment of the anatomy enables the establishment of a treatment strategy ahead of time, and results in increased time efficiency and operator confidence during the treatment procedure [4].

In our centre, a standardized triphasic liver CT protocol encompassing a late arterial, portal venous and equilibrium phase had been used to evaluate the liver parenchyma and vasculature in all patients with liver malignancies, including radioembolisation candidates [4–6]. The imaging delay of the arterial phase (post-threshold delay of 20 s) was chosen to allow for detection of hypervascular tumours, but it is questionable whether a late arterial phase is best suited for evaluation of the arterial vasculature.

We noticed in clinical practice that contrast enhancement of the portal vein often obscures the origin of small arterial branches that need to be identified, especially the right gastric artery (RGA) and segment 4 artery (A4). An early arterial phase with a delay of 10 s may reveal the RGA and A4 origin better due to a higher contrast-to-noise ratio (CNR) of the hepatic artery relative to the portal vein. Furthermore, it remains uncertain how well the RGA and A4 origins can be visualized on liver CT in the population of heavily pretreated radioembolisation patients, and how reproducible these observations are when comparing different raters.

Hence, the purpose of this study was to compare RGA and A4 origin detection rates during radioembolisation workup between early and late arterial phase liver CT protocols, and to determine inter-rater reproducibility.

## Methods

### Study design

We performed a prospective development study in accordance with the IDEAL recommendations [7]. A triphasic liver CT (henceforth referred to as standard protocol) was already part of our routine workup for radioembolisation. The majority of our radioembolisation candidates have hypovascular tumour types. Thus, evaluation of the hepatic arterial anatomy is the primary purpose of the arterial liver CT phase in these patients, while the portal venous phase is used for tumour detection and localization. To improve the visualization of small arterial branch origins, we adjusted our liver CT protocol (henceforth referred to as adjusted protocol). The performance of the adjusted scan protocol was tested by using a historical cohort of patients previously scanned with the standard protocol as comparison.

The medical ethics committee of our institution approved this study and waived the need for informed consent for reviewing imaging data on radioembolisation patients in our centre.

### Study population

A total of 100 patients (50 patients for each of the protocols) would be required to demonstrate a 30 % difference in RGA detection rate (80 % for the adjusted protocol vs. 50 % for the standard protocol) with a power of 0.90, at an alpha-level of 0.05 (sample size calculation with ‘Power and Sample Size Calculation’ version 3.1.2 for MacOsX).

All patients with unresectable and chemorefractory liver tumours who underwent a pretreatment liver CT in combination with an ^18^F-FDG-PET scan on the same CT scanner, before undergoing a preparatory angiography as part of the radioembolisation workup, were eligible for study participation. Starting in December 2013, 50 consecutive patients were selected for the adjusted liver CT protocol. A group of 50 consecutive patients who had been scanned earlier using the standard protocol was selected for comparison. Patients with a technical scan failure or those without a preparatory angiography were excluded. Baseline patient characteristics for both groups were compared to ensure a valid comparison.

### Technique, equipment and scan settings

On the day before the examination, all patients received 1,000 ml of water-soluble contrast solution (Telebrix Gastro 300 mg/ml) orally to enhance the detection of peritoneal disease and lymphadenopathy. In the adjusted protocol, mannitol solution (100 ml, 15 %) was given orally before the scan to stimulate gastric emptying and create a negative contrast with adjacent contrast-enhanced vessels. Subsequently, 150–185 ml (depending on body weight) iopromide 300 mg/ml contrast agent (Ultravist, Bayern Schering Pharma AG, Berlin, Germany) was injected with a double syringe injector in the antecubital vein at a rate of 5 ml/s, followed by a 50-ml NaCl chaser.

All CT scans were acquired under breath-hold on an integrated PET/CT scanner (Biograph mCT, Siemens Healthcare, Erlangen, Germany) with 40 detector rows, using a matrix size of 512 × 512, rotation time of 0.4–0.5 s, pitch of 0.9–1.2, and fixed 120 kV tube potential.

The *standard protocol* consisted of a late arterial, portal-venous and an equilibrium phase, which were obtained with a post-threshold (abdominal aorta enhancement >100 Hounsfield units (HU)) delay of 20, 55 and 300 s, respectively. A tube current of 150 mAs was used in the arterial and equilibrium phase, and 225 mAs for the portal-venous phase. Slice thickness/increment were 0.9/0.7 cm for the arterial phase, and 1.5/1.0 cm for the portal-venous and equilibrium phase.

The *adjusted protocol* consisted of an arterial phase with a shortened post-threshold delay of 10 s, and an unchanged timing of the portal-venous phase. No equilibrium phase was acquired.

In this protocol, tube current was dependent on the weight of the patient: 150 mAs in patients <85 kg, and 200 mAs in patients ≥85 kg. In all phases, slice thickness/increment was 0.9/0.7 mm.

The technique used during the preparatory angiography has been published before and conforms to current standards of clinical practice [8, 9]. Digital subtraction angiography (DSA) images were acquired on an Allura Xper FD20 system (Philips, Best, The Netherlands).

### Image analyses

All images were anonymized and loaded into OsiriX (version 5.8, 32-bit, MacOS X) for image analyses.

Two independent raters, an abdominal radiologist (MB) and an interventional radiologist (PJvD), were asked to score the following: visibility of the RGA origin (yes/no), location of the RGA origin (arrow appointing the region of interest), visibility of the A4/MHA origin (yes/no), location of the A4/MHA origin/origins (arrow), and ability to distinguish two separate branches to S4a and S4b (yes/no). The raters were instructed to score the origin as not visible when in doubt.

The origins were evaluated on the thin slices (1 mm thickness/0.7 mm increment). Use of a maximum intensity projection (MIP) with a reconstructed slice thickness of 4 mm and/or the use of multiplanar reconstruction was optional. Scans of patients who had previously undergone surgery in which the RGA was sacrificed or segment 4 was resected were considered non-evaluable.

The A4 was termed a middle hepatic artery (MHA) when originating in between a (r)left hepatic artery (LHA) and (r)right hepatic artery (RHA), as a branch from the common hepatic artery (CHA) or proper hepatic artery (PHA). No true PHA exists in patients with an aberrant hepatic artery, therefore the RGA or A4 origin was called after the non-aberrant arterial branch if originating distal to the origin of the gastroduodenal artery (GDA) [4].

A third rater (AvdH) independently scored the origin of the RGA on DSA to establish a reference standard for correct identification of the RGA origin on CT. This was not done for the A4 score, since the lack of topographical landmarks on DSA makes distinguishing liver segments challenging. Unfortunately, C-arm CT images were not available in all patients. The third rater also assessed the individual hepatic arterial anatomy as described earlier [4], and measured the signal-to-noise ratio (SNR) of the hepatic arteries and portal veins, as well as the CNR of the hepatic arteries relative to the portal vein. Circular regions of interest (ROIs) with a diameter of 3–6 mm were drawn in the hepatic artery and the portal vein at approximately the same level in the liver hilum on axial slice MIP (8-mm reconstructed slice thickness) images of the arterial phase, and the mean and standard deviation (SD) of the signal (in HU) were noted. The following equations were used:

1 $$ SNR=\frac{Mean\ (HU)}{SD(HU)} $$

2 $$ CNR=\frac{Mea{n}_{arteries}-Mea{n}_{portal\  vein}}{\sqrt{\raisebox{1ex}{$1$}\!\left/ \!\raisebox{-1ex}{$2$}\right.*\left(S{D_{arteries}}^2+S{D_{portal\  vein}}^2\right)}} $$

### Statistical analysis

Descriptive analyses were performed to give an overview of baseline patient characteristics, and RGA/A4 origin locations. Data with normal and non-normal distributions are presented as mean ± standard deviation and median (range).

A two-sided unpaired Student’s t-test was used to test for differences in mean arterial and portal SNR, and mean CNR, between the standard and the adjusted protocol.

A chi-square test was used to compare the standard protocol and the adjusted protocol with regard to the rate of correct RGA origin localization (using DSA as a reference standard), and A4 origin detection.

A specific proportion of agreement and kappa statistics were used to indicate inter-rater agreement and reliability for the RGA and A4 origin detection.

All analyses were performed with R Studio version 0.98.1102 for MacOsX. A p-value <0.05 was considered statistically significant.

## Results

### Patients and scans

Between December 2013 and January 2015, 58 patients were scanned according to the adjusted protocol for liver CT, and were found eligible for inclusion in this study. Eight of these patients were excluded either because the scan was performed on another CT scanner (n = 6) or because no angiography was performed (n = 2). Between May 2012 and December 2013, 63 patients had undergone a standard triphasic liver CT. Twelve of these patients were not selected for the control group because the scan was acquired on another CT scanner (n = 9), no angiography was performed (n = 2) or technical failure of the CT scan occurred (n = 1). The two groups were comparable with regard to baseline patient characteristics and hepatic arterial anatomy (Tables 1 and 2).

**Table 1:** Baseline patient characteristics

Characteristic	Entire cohort (n = 100)	Standard protocol (n = 50)	Adjusted protocol (n = 50)
Age, years	64 ± 10	63 ± 11	65 ± 11
Gender			
Male	54 (54 %)	24 (48 %)	30 (60 %)
Female	46 (46 %)	26 (52 %)	20 (40 %)
BMI	27 ± 4	27 ± 5	27 ± 4
Liver tumour burden			
<25 %	83 (83 %)	42 (84 %)	41 (82 %)
25–50 %	12 (12 %)	6 (12 %)	6 (12 %)
>50 %	5 (5 %)	2 (4 %)	3 (6 %)
Liver tumour type			
CRC	66 (66 %)	31 (62 %)	35 (70 %)
Cholangiocarcinoma	11 (11 %)	6 (12 %)	5 (10 %)
Uvea melanoma	8 (8 %)	4 (8 %)	4 (8 %)
Mammaca	5 (5 %)	3 (6 %)	2 (4 %)
Other	10 (10 %)	6 (12 %)	4 (8 %)
Previous surgery involving			
A4	6 (6 %)	2 (4 %)	4 (8 %)
RGA	5 (5 %)	4 (8 %)	1 (2 %)

The baseline patient characteristics are summarized in this table, for the entire cohort and for both protocols separately

Values are given as mean ± standard deviation or number of patients (percentage of total)

*BMI* body mass index, *CRC*, *RGA* right gastric artery

**Table 2:** Individual hepatic arterial anatomy

Anatomical variant	Entire cohort (n = 100)	Standard protocol (n = 50)	Adjusted protocol (n = 50)
Standard anatomy	54 (54 %)	29 (58 %)	25 (50 %)
Early branching pattern	19 (19 %)	9 (18 %)	10 (20 %)
Early branching LHA	2 (2 %)	1 (2 %)	1 (2 %)
Early branching RHA	1 (1 %)	0	1 (2 %)
Trifurcation of CHA	13 (13 %)	7 (14 %)	6 (12 %)
Quadrifurcation of CHA	3 (3 %)	1 (2 %)	2 (4 %)
Aberrant hepatic arteries	27 (27 %)	12 (24 %)	15 (30 %)
rLHA [LGA, S2-3]	8 (8 %)	2 (4 %)	6 (12 %)
rLHA [LGA, S2-4]	2 (2 %)	1 (2 %)	1 (2 %)
aLHA [LGA, S2]	2 (2 %)	1 (2 %)	1 (2 %)
rRHA [SMA, S5-8]	9 (9 %)	5 (10 %)	5 (10 %)
rRHA [SMA, S4-8]	0	0	0
aRHA [SMA, S5+8]	1 (1 %)	0	1 (2 %)
rLHA + rRHA [SMA, S5-8]	1 (1 %)	0	1 (2 %)
rCHA [SMA]	4 (4 %)	3 (6 %)	1 (2 %)

The hepatic arterial configuration is summarized in this table, for the entire cohort and for both protocols separately

Values are given in number of patients (percentage of total)

Aberrant hepatic arteries are indicated as type of variant [origin, segmental vascularisation pattern]

*LHA* left hepatic artery, *RHA* right hepatic artery, *CHA* common hepatic artery, *SMA* superior mesenteric artery

### Origin of the right gastric artery (RGA)

**Fig. 1:**
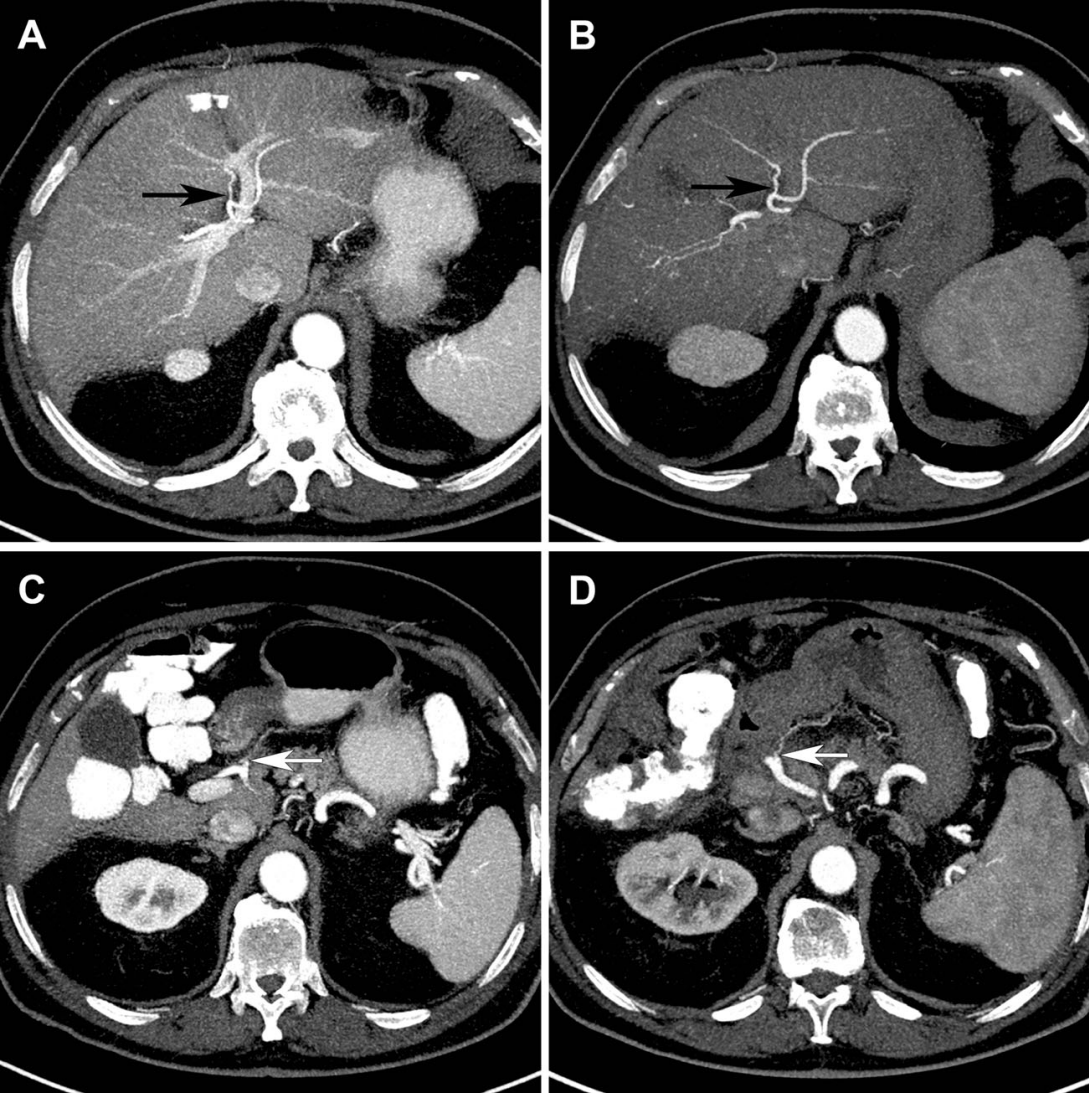
**a**-**d** Comparison of maximum intensity projections of liver CT images with the standard (**a**, **c**) and the adjusted arterial phase protocol (**b**, **d**) in the same patient, acquired on the same scanner. Note that the adjusted protocol is easier to evaluate due to an increased contrast-to-noise ratio for the hepatic artery relative to the portal vein, but the origins of the A4 (**a**, **b**, black arrow) and the right gastric artery (**c**, **d**, white arrow) are nonetheless visible in both protocols

Five scans (standard protocol n = 4, adjusted protocol n = 1) were not evaluable for the assessment of the RGA origin, due to previous gastric (n = 4) or pancreatic (n = 1) surgery involving the RGA, leaving 46 scans evaluable for the standard protocol and 49 scans for the adjusted protocol (see Fig. 1 for a clinical example of both protocols).

The RGA origin was visible in 70/95 patients (74 %) on DSA, and in 55/95 (58 %) and 62/95 (65 %) on CT for raters 1 and 2, respectively (Table 3). This corresponds to the following test characteristics for CT, taking DSA as a reference standard (range for raters 1–2): positive-predictive value 84–85 %, negative-predictive value 40–48 %, false-positive rate 36–36 %, false-negative rate 24–34 %. Thus, both raters recognized the RGA origin on CT in nine of the 25 (36 %) patients in whom it was not visible on DSA.

**Table 3:** Origin detection of the right gastric artery (RGA)

	DSA (n = 95)	CT rater 1 (n = 95)	CT rater 2 (n = 95)
Origin of RGA visible?			
Yes	70 (74 %)	55 (58 %)	62 (65 %)
No	25 (26 %)	40 (42 %)	33 (35 %)
RGA origins			
CHA	6 (9 %)	8 (15 %)	7 (11 %)
GDA	3 (4 %)	5 (9 %)	3 (5 %)
PHA	18 (26 %)	11 (21 %)	15 (24 %)
LHA	35 (49 %)	26 (47 %)	29 (47 %)
A4/MHA	3 (5 %)	2 (4 %)	2 (3 %)
RHA	5 (7 %)	3 (6 %)	5 (8 %)
SMA	0	0	1 (2 %)
	Standard protocol (n = 46)	Adjusted protocol (n = 49)	
RGA origin correctly identified on CT (DSA as reference test)			
Rater 1	25 (54 %)	34 (69 %)	
Rater 2	30 (65 %)	34 (69 %)	

The RGA origin and its detection rate is summarized for DSA and CT (both raters) in this table

Values are given in number of patients (percentage of total)

No true PHA exists in patients with an aberrant hepatic artery, therefore, the RGA was called after the non-aberrant arterial branch if originating distal to the origin of the GDA

*DSA* digital subtraction angiography, *CHA* common hepatic artery, *GDA* gastroduodenal artery, *PHA* proper hepatic artery, *LHA* left hepatic artery, *MHA* middle hepatic artery, *RHA* right hepatic artery, *SMA* superior mesenteric artery

The RGA origin was correctly localized on CT (taking DSA as reference standard) by rater 1 in 54 % and 69 % of patients scanned with the standard and adjusted protocols, respectively (*p* = 0.19); for rater 2, this was 65 % and 69 %, respectively (*p* = 0.83).

The specific proportion of agreement in CT scores between the two raters was 78/95 (82 %), and the reliability was substantial with a Cohen’s kappa of 0.62 (confidence limits 0.46–0.78). Both raters appointed the same RGA origin in all cases with a visible RGA on CT. According to the CT scores of raters 1–2, the RGA originated from the LHA in 47–51 % of patients, PHA in 21–23 %, CHA in 10–13 %, GDA in 5–9 %, RHA in 6–8 %, A4/MHA in 3–4 % and superior mesenteric artery (SMA) in 2 %.

### Origin of the A4/middle hepatic artery (MHA)

Six scans (standard protocol n = 2, adjusted protocol n = 4) were not evaluable for the assessment of the A4/MHA origin, due to previous liver surgery involving segment 4, leaving 48 scans evaluable for the standard protocol and 46 scans for the adjusted protocol.

The first and second rater scored the origin of the A4/MHA as visible in 90/94 (96 %) and 84/94 (89 %), respectively (Table 4). The detection rate of the A4/MHA origin did not differ significantly between the standard and the adjusted protocol: 96 % versus 96 % scored by rater 1, and 87 % versus 91 % (*p* = 0.79) scored by rater 2.

**Table 4:** Origin detection of the A4/middle hepatic artery (MHA) on CT

	Entire cohort (n = 94)	Standard protocol (n = 48)	Adjusted protocol (n = 46)
Origin of A4/MHA visible?			
Rater 1			
Yes	90 (96 %)	46 (96 %)	44 (96 %)
No	4 (4 %)	2 (4 %)	2 (4 %)
Rater 2			
Yes	84 (89 %)	42 (87 %)	42 (91 %)
No	10 (11 %)	6 (13 %)	4 (9 %)
A4/MHA origins			
Rater 1			
CHA	4 (4 %)	1 (2 %)	3 (7 %)
PHA	3 (3 %)	3 (7 %)	0
LHA	51 (57 %)	28 (61 %)	23 (52 %)
rLHA	2 (2 %)	1 (2 %)	1 (2 %)
RHA	25 (28 %)	13 (28 %)	12 (27 %)
rRHA	0	0	0
LHA + RHA	4 (4 %)	0	4 (9 %)
rLHA + RHA	1 (1 %)	0	1 (2 %)
Rater 2			
CHA	4 (5 %)	1 (2 %)	3 (7 %)
PHA	4 (5 %)	4 (10 %)	0 (0 %)
LHA	47 (56 %)	26 (62 %)	21 (50 %)
rLHA	1 (1 %)	1 (2 %)	0
RHA	23 (27 %)	10 (24 %)	13 (31 %)
rRHA	0	0	0
LHA + RHA	4 (5 %)	0	4 (10 %)
rLHA + RHA	1 (1 %)	0	1 (2 %)

The A4/MHA origin and its detection rate are summarized for both raters in this table, for the entire cohort and for both protocols separately

Values are given in number of patients (percentage of total)

*CHA* common hepatic artery, *PHA* proper hepatic artery, *LHA* left hepatic artery, *RHA* right hepatic artery

The specific proportion of agreement in visibility of the A4/MHA origin between the two raters was 88/94 (94 %). The reliability was moderate; Cohen’s kappa was 0.54 (confidence limits 0.23–0.86). There was a disagreement between the two raters in the appointed A4/MHA origin location on CT in four patients.

According to raters 1–2, segment 4 was vascularized exclusively by an A4 originating from the LHA in 56–57 % of patients, from the RHA in 27–28 %, from a rLHA in 1–2 % and never from a rRHA. An MHA originated from the CHA in 4–5 % of patients, and from the PHA in 3–5 %. A dual-type A4 originated from the LHA + RHA in 4–5 % of patients, and from a rLHA + RHA in 1 %.

Raters 1 and 2 indicated that they could clearly distinguish two separate arterial branches to the superior (S4a) and inferior (S4b) parts of S4 in 18/94 (19 %) and 10/94 (11 %) of patients. These two branches originated from a different main hepatic arterial branch (LHA + RHA or rLHA + RHA) in five of these patients, and originated from the same main hepatic arterial branch in the rest of the cases.

### Signal-to-noise (SNR) and contrast-to-noise (CNR) ratios

The mean arterial SNR was not different for the two protocols: 7.5 and 7.4 for the adjusted protocol and the standard protocol, respectively (95 % confidence interval (CI) for the difference in means: −1.2 to 1.5; *p* = 0.83). The mean portal SNR was significantly lower in the adjusted protocol compared with the standard protocol: 7.4 versus 9.7 (95 % CI for the difference in means: −3.2 to −1.3; *p* = 8.9 × 10^-6^). This resulted in a significantly higher mean CNR of the hepatic arteries relative to the portal vein in the adjusted protocol: 1.7 versus 1.2 (95 % CI for the difference in means: 0.4–0.7; *p* = 4.9 × 10^-11^).

## Discussion

The purpose of this study was to compare RGA and A4/MHA origin detection rates during radioembolisation workup between early and late arterial phase liver CT protocols, and to determine inter-rater reproducibility. The adjusted protocol with the early arterial phase did not significantly improve the detection rate of small intra- and extrahepatic branches, despite a higher CNR of the hepatic arteries relative to the portal vein. Furthermore, the RGA origin detection was lower than that of the A4 (58–65 % vs. 89–96 %) in our study. However, when identified, the inter-rater agreement of the origin localization was high for both the RGA (82 %) and A4 (94 %). The inter-rater reliability was moderate and substantial, respectively.

The overall RGA origin detection rate on CT (around 60 %) can be considered suboptimal. By comparison with DSA, we found a low negative-predictive value (40–48 %) and relatively high false-negative rate (24–34 %), indicating that the inability to find the RGA origin on CT does not signify the same for DSA. The RGA origin may not be visualized on CT because of a small calibre, an intimate anatomical course parallel to another branch, flow dynamics prohibiting contrast filling or the presence of metal artefacts. The high positive-predictive value (84–85 %), and good inter-rater agreement, on the other hand, suggest that the necessity to coil-embolize the RGA or advance the microcatheter beyond its origin to avoid harmful extrahepatic deposition of radioactive microspheres, can already be evaluated in patients with a visible origin of the RGA on pretreatment liver CT. Besides, the RGA origin was revealed by CT in 36 % of the patients in whom DSA failed to show it. Thus, assessing the RGA origin on pretreatment liver CT remains beneficial.

The A4/MHA origin detection rate is much higher, which has several important benefits. Depending on the hepatic arterial configuration of an individual patient, it can be decided to use the A4/MHA as a separate site of administration, include it in a more proximal injection position or coil-embolize it to induce intrahepatic redistribution of blood flow [4, 10]. Furthermore, it allows pretreatment activity calculations to be performed with knowledge of the segment 4 vascularisation. This is crucial to avoid under- or overdosing of segment 4 when performing radioembolisation on a single-session basis, and it may contribute to a more reliable distribution of ^99m^TC-macroaggregated albumin (^99m^Tc-MAA) during a routine pretreatment procedure [11, 12]. Interestingly, we found that the typical distinction of segment 4 into an upper (4a) and lower (4b) segment as based on the portal vascularisation, could only be made in 11–19 % of patients.

The radiologists in our centre prefer the early arterial phase for vascular evaluation due to the increased ease of use associated with the higher CNR on maximum intensity projections. It should, however, be noted that the 10-s delay can be too short to allow for optimal enhancement of hypervascular tumours. In these patients, the use of an early and late arterial phase (10- and 20-s post-threshold delay, respectively) may be considered if no other imaging is available to substitute tumour evaluation.

C-arm CT has vastly improved the possibilities for intra-procedural imaging. It allows for 3D-imaging of contrast-enhanced vessels in relation to surrounding soft tissue, and we have previously demonstrated that C-arm CT is capable of showing the intra- and extrahepatic arterial perfusion territory of target branches during radioembolisation workup [13]. It may, therefore, be regarded as the new gold standard for vascular evaluation. Unfortunately, we could not use C-arm CT as a reference standard because it was not available in all patients treated before 2013. We used DSA instead for the RGA origin detection. This was deemed unfeasible for the A4 origin detection, due to challenges posed by overprojection of intrahepatic branches on the 2D DSA-images and lack of soft-tissue landmarks. It should be noted that the excellent capacities of C-arm CT do not render optimization of pre-procedural imaging useless. An accurate assessment of the hepatic arterial anatomy before the preparatory angiography enables discussing and defining a treatment strategy ahead of time, and increases time-efficiency as well as operator confidence during the preparatory angiography [4].

Our study had several limitations. First, our sample size was based on an assumption of a reasonable number of patients to compare the two protocol groups, because specific detection rates for this study population were lacking. However, baseline characteristics were comparable. Furthermore, we allowed raters to use both MIP and non-MIP images to detect the RGA and A4 origins. For CNR assessment, we used MIP images only. We believe that this has contributed to the difference in results for the CNR measurements and origin detection assessments. In clinical practice radiologists are, however, not restricted to a specific method to evaluate images. In addition, we tried to blind the raters for the type of protocol used in a scan, but because the difference in CNR was so explicit and the gastric preparation was slightly different in the two protocols, they could still differentiate between the two protocol types. Finally, we only evaluated the origin of the RGA and A4, because these branches are present in all patients and have important implications. However, in some patients other small arterial branches are also important, such as the artery to segment 1, extrahepatic branches to the pancreas or duodenum, and parasitized extrahepatic arteries. Further research needs to clarify whether these branches and their origin can be visualized on pretreatment CT.

CT hardware and acquisition protocols are continually evolving. In this study, we focused on the arterial phase delay timing, but changes in other technical parameters may lead to further improvements of the liver CT acquisition protocol. The use of a higher contrast agent concentration and injection rate, a patient-tailored scan delay based on a test bolus of contrast agent, and scanning at lower energy levels are among the most promising developments [6, 14, 15].

## Conclusion

A 10-s delay arterial phase protocol does not significantly improve the detection rate of small intra- and extrahepatic branches, despite an increased CNR of the hepatic arteries relative to the portal vein. Ease of use with this protocol needs to be weighed against the lesser sensitivity for hypervascular tumour detection. The RGA origin detection rate is currently suboptimal, whereas the A4/MHA origin detection rate is much higher, with good inter-rater reproducibility. Nevertheless, CT remains important for preprocedural planning, because it may reveal arterial anatomy not discernible on DSA.

## Abbreviations

aLHA/aRHA: Accessory LHA/RHA
CHA: Common hepatic artery
CNR: Contrast-to-noise ratio
CECT: Contrast-enhanced computed tomography
DSA: Digital subtraction angiography
GDA: Gastroduodenal artery
LHA: Left hepatic artery
CT: Computed tomography
MHA: Middle hepatic artery
PHA: Proper hepatic artery
rCHA: Replaced CHA
RGA: Right gastric artery
RHA: Right hepatic artery
rLHA/rRHA: Replaced LHA/RHA
A4: Segment 4 artery
SD: Standard deviation
SNR: Signal-to-noise ratio
SPA: Specific proportion of agreement
